# *Hypericum perforatum* to improve post-operative Pain Outcome after monosegmental Spinal microdiscectomy (HYPOS): a study protocol for a randomised, double-blind, placebo-controlled trial

**DOI:** 10.1186/s13063-018-2631-6

**Published:** 2018-04-25

**Authors:** Christa Raak, Wolfram Scharbrodt, Bettina Berger, Arndt Büssing, René Geißen, Thomas Ostermann

**Affiliations:** 1 Institute of Integrative Medicine, Witten/Herdecke University, 58313 Herdecke, Germany; 2 Integrative Neuromedicine, Community Hospital Herdecke, Witten/Herdecke University, 58313 Herdecke, Germany; 3 Chair of Research Methodology and Statistics in Psychology, Witten/Herdecke University, 58448 Witten, Germany; 4 Centre for Clinical Trials, Witten/Herdecke University, 58448 Witten, Germany

**Keywords:** Lumbar microdiscectomy surgery, *Hypericum perforatum*, Post-operative pain, Homeopathy, Study protocol

## Abstract

**Background:**

Spinal disc herniation is a frequently occurring degenerative disease of the spine. Many patients undergoing surgery suffer from radicular pain, known as memory pain, beginning from the third post-operative day. This results in the prescription of high-dose opioid medications. In homeopathy, *Hypericum perforatum* is known as a remedy for unbearable, shooting or jabbing pain especially when neural damage is involved. Reduction of pain after application of *H. perforatum* has been observed in previous studies. This study is aimed to investigate whether homeopathic *H. perforatum* in a potentisation of C200 leads to the reduction of post-operative pain and a decrease of pain medication compared to placebo.

**Methods/design:**

This is a monocentric, double-blind, randomised placebo-controlled trial conducted at the Department of Neurosurgery at the Community Hospital Herdecke, Germany. One hundred study participants are being recruited from inpatients undergoing elective, monosegmental, lumbar microdiscectomy surgery. Patients are randomly allocated to receive homeopathic treatment or placebo in addition to usual pain management after surgery. The primary clinical outcome is pain reduction after 3 days of inpatient care as measured by pain reduction of subjective pain on a 100-mm Visual Analogue Scale (VAS) at the third post-operative day. Statistical analysis will be carried out by means of a covariance model with adjustment for baseline values and patient expectation for all randomised patients.

**Discussion:**

This study is the first trial of classical homeopathy that will evaluate the efficacy of homeopathic *H. perforatum* after monosegmental spinal microdiscectomy. We intend to clarify the potential of homoeopathic *H. perforatum* to reduce surgery-associated pain.

**Trial registration:**

German Clinical Trials Register, ID: DRKS00007913. Registered on 17 October 2014.

EudraCT – Nr: 2013–001383-31.

Data sets from the German Clinical Trials Register (DRKS, Deutsches Register Klinischer Studien) are updated every 4 weeks automatically to the International Clinical Trials Registry Platform of World Health Organisation: http://apps.who.int/trialsearch/.

**Responsibilities**

Sponsor:

Witten/Herdecke University

Alfred-Herrhausen-Straße 50

58,448 Witten

Deputy of the sponsor:

Dr. Wolfgang Eglmeier (Head of Centre for Clinical Trials Witten/Herdecke)

Alfred-Herrhausen-Straße 50

58,448 Witten

E-mail: wolfgang.eglmeier@uni-wh.de

Principal investigator:

Prof. Dr. med. Wolfram Scharbrodt

Community Hospital Herdecke

Department for Neurosurgery

Gerhard-Kienle-Weg 4

58,313 Herdecke

w.scharbrodt@gemeinschaftskrankenhaus.de

Project coordination:

Christa Raak

Faculty for Health (Department for Integrative and Anthroposophic Medicine)

University Witten/Herdecke gGmbh

Gerhard-Kienle-Weg 4

58,313 Herdecke

christa.raak@uni-wh.de

Project manager/data analysis/biometry:

Prof. Dr. Thomas Ostermann

Faculty for Health (Department for Psychology and Psychotherapy)

University Witten/Herdecke gGmbh

Alfred-Herrhausen-Straße 50

58,448 Witten

thomas.ostermann@uni-wh.de

**Electronic supplementary material:**

The online version of this article (10.1186/s13063-018-2631-6) contains supplementary material, which is available to authorized users.

## Background

Disc herniation is one of the most common degenerative diseases of the lumbar spine. The incidence for lumbar disc herniation in Germany is 150/100,000 people per year. Sixty out of those 100,000 patients undergo intervertebral disc surgery, resulting in 50,000 operations a year [1]. The prevalence of a herniated disc is estimated to be 20–30% for the age group between 35 and 64 years. In a review of the prevalence of degenerative lumbar disc lesions, Battié et al. reported frequencies for a reduced disc height of 3–56% and for lumbar herniation of 3–63% depending on the design of the study [2]. Moreover, it was noted that only very few studies investigated the frequency of disc herniation with segment-related radiculopathy at all. The results show that the frequency for this incident is in the low single-digit range [3].

Treatment options for a lumbar disc herniation are determined by the duration and presence of the symptoms and the severity of the resulting pain. Besides, the nature of the symptoms (such as weakness or numbness), the patient’s age and further progression of pain relief are important decision-making parameters for conservative therapy or surgery [4]. If medication, physical therapy, and/or other non-surgical treatments have not led to a significant symptom improvement over 6 weeks, a relative indication for surgery is given [4]. Patients who undergo lumbar surgery as well as patients who are not operated on both improve within 2 years. However, there is a trend with regard to pain reduction in favour of patients who undergo lumbar disc herniation surgery [5]. This is underpinned by the 4-year results of the Spine Patient Outcomes Research Trial (SPORT) which showed significantly better results in the pain scores in surgical patients compared to the conservatively treated group [6].

Depending on the main diagnosis, in 2010, patients with lower back problems were subjected to back surgery in 38% of the cases [7]. Since the introduction of the Diagnosis Related Groups (DRG), the length of stay in Germany after minimal invasive surgery is about 3–4 days if no adverse events (AEs) occur, which is comparable with international data [8].

For the feasibility and the course of mobilisation and rehabilitation, post-surgical pain management plays a crucial role [9]. The post-surgical phase requires adequate pain therapy according to the World Health Organisation (WHO) analgesic ladder. Non-opioid analgesics (ibuprofen, diclofenac, novaminsulphon, step 1) and weak opioids (tilidine or tramadol, step 2) are most commonly used. Only in very rare cases are strong opioids (step 3) applied [4, 10]. Because of possible side effects, i.e. gastrointestinal pain, sedation, peptic ulceration, allergy and rash, respiratory depression, nausea and vomiting, rash and urinary retention, the choice of the non-steroidal anti-inflammatory drugs (NSAIDs) and opioids must be individually tailored to the patient, aiming to control inflammation and its consequent pain [9–12].

At the Community Hospital Herdecke, Germany, homeopathic remedies are also offered in addition to the standard treatments mentioned above on patient request [13]. Homeopathy is one of the oldest traditional European medical systems founded by the German physician Samuel Hahnemann (1755–1843) in 1796. It is based on ‘the principle of similars’ already described in ancient Greek sources of medicine. According to the ‘like cures like’ principle of healing, diluted substances that cause symptoms in healthy individuals can be used to treat patients with similar symptoms [14]. A homeopathic remedy is made by a process of dilution and succession. They are named by their grade of dilution as decimal (D), centesimal (C) or quinquagintamillesimal (Q or LM) potencies [15]. Thus, a dilution of D6 indicates that the original substance is diluted by a factor of 10^-6^, while a dilution of C30 denotes a dilution of 100^-30^ [16]. Homeopathic remedies can be prescribed individually, but patients are often treated with typical remedies that have shown to be effective according to clinical experience, irrespective of the individuality of a given patient [17]. This idea of the concept of ‘proven indications’ was made famous by the Austrian physician Mathias Dorcsi in the twentieth century [18].

From a homeopathic perspective, the administration of *Hypericum perforatum* is indicated in order to improve wound healing and reduce repeatedly occurring neural pain [19, 20].

*H. perforatum* (St. John’s wort) is a member of the *Guttiferae* family with about 400 known species of *Hypericum* in Europe and is one of the oldest medical plants with a history of more than 2000 years. It grows in sunny locations with well-drained, limey soil and reaches a height of 50 to100 cm. Yellow star-shaped flowers, often clustered in a trio, have five petals and the leaves contain tiny, transparent oil glands resembling perforations [21].

Experimental studies have demonstrated that the administration of potentiated *Hypericum* can lead to a reduction of symptoms due to spinal disc pathology and associated pain caused by a pinched and irritated nerve [22]. In clinical trials the pain-reducing effect of *Hypericum* could also be observed in a number of studies, amongst others in neuropathic pain [23–25], after reconstruction of knee ligaments [26], or after tooth extraction [21]. However, this effect was only shown in combination with other homeopathic remedies. The question to what extend the results shown in these studies can be attributed to the specific effects of *Hypericum* have not been resolved so far. The adjuvant administration of *Hypericum* for the present indication of post-surgical pain after lumbar microdiscectomy surgery has not been investigated within the framework of a scientific clinical setting.

### Aims

The aim of the study is to investigate the effect of *Hypericum* C200 in post-operative pain management. The primary aim of the study is to assess the reduction of subjective pain on the 100-mm VAS at the third post-operative day. In consideration of the side effects of conventional pain medication and the potential benefit of *Hypericum* in wound healing and neural pain reduction, it is reasonable also to investigate the dosage of conventional analgesic intake between the groups as a secondary outcome measure.

Finally, the reduction of subjective pain on the VAS at the fifth post-operative day and the development of sensory and affective pain perception (as assessed using the Pain Experience Scale ‘Schmerzempfindungsskala’ (SES)) between the groups, as well as the amount of post-surgical intake of analgesic/anti-inflammatory agents, are also secondary outcomes.

## Methods/design

### Study design

The study will be a randomised, placebo-controlled, double-blind trial with a 3–5-day duration.

### Study setting

The study will be conducted at the Department of Neurosurgery at the Community Hospital Herdecke, Germany.

The Community Hospital Herdecke is a hospital for acute cases requiring supportive care level II with specific responsibility for ambulant and outpatient care. It contains over 471 beds and around 50,000 patients are treated here yearly, ambulant or as outpatients. For a number of years the hospital has offered an established integrative therapy concept with the emphasis on holistic/anthroposophic medicine. It is an academically specialised hospital and has a research centre with methodological competence in the areas of basic research, clinical research and research in supportive care [27].

Over 900 brain and spinal surgeries, or surgeries involving peripheral nerves, are carried out at the department of neurosurgery each year.

### Participant recruitment

Participant recruitment precedes allocation, meaning that participants will be recruited from those electively admitted to the Department of Neurosurgery at the Community Hospital Herdecke for monosegmental spinal microdiscectomy surgery due to a lumbar disc herniation. They will be approached by the responsible neurosurgeon and made aware of the ongoing study within the framework of a counselling interview. No further recruiting measures will be taken. After signing the informed consent, patients will be allocated to one study arm. No study participants will be included who are in a direct relationship with, or dependent on, the sponsor or research team (students, employees of the institution, close relatives).

### Eligibility criteria

The suitability of a patient will be verified pre-operatively based on the following inclusion criteria:

- ➢ Clinical indication for the described monosegmental spinal microdiscectomy
- ➢ Age between 18 and 85 years
- ➢ Informed consent/declaration of data protection
- ➢ Legal capacity

In case one of the following criteria holds for a patient they will be excluded from the study:

- ➢ Neurosurgical intervention requirement
- ➢ Previous diagnosis of a somatic pain condition
- ➢ Ongoing early retirement proceedings due to back problems
- ➢ Other chronic pain conditions not caused by a lumbar spinal stenosis
- ➢ Gravid or breast-feeding women
- ➢ Limited communicative ability
- ➢ Consumption of sedative medications
- ➢ Consumption of other homeopathic remedies
- ➢ Severe comorbidities
- ➢ Acute psychotic conditions
- ➢ Simultaneous participation in other clinical studies or completion of participation in a study less than 6 months prior (patients are explicitly asked about this)
- ➢ Ongoing accommodation in an institution after official or judicial order

### Intervention

There will be no restriction in terms of post-operative standard treatment and on-demand pain medication in any way. Homeopathic remedies will be applied as an add-on with a time delay of at least 30 min after food intake or alongside oral hygiene. Regarding dosage, the present study is orientated on the dosage recommendations of the Drug Commission D of the German Medical Associations for homeopathic remedies in high grades of dilution [28] as well as the dosages of comparative clinical studies of Frass et al. [29, 30] and Paris et al. [26], who applied a similar therapy scheme in their studies. Patients will either receive three globules of *H. perforatum* C200 or placebo twice a day every 12 h. The application of homeopathic remedies will start in the morning of the first post-operative day up to the fifth post-operative day.

The intake of the study medication will be monitored and is documented using the number of returned empty pharmaceutical vials which contained either placebo or the active treatment.

### Criteria for discontinuing or modifying allocated interventions

Patients can be excluded from further participation within the study or withdraw themselves without having to provide any further reasons. Possible reasons for this therapy dropout are expected to be:

- ➢ Withdrawal of the patient’s consent
- ➢ Consumption of drugs or, rather, medications which are not permitted during the duration of the clinical study
- ➢ Deficient compliance of patients after the evaluation of the examining physician (regular and specified consumption of study medication)
- ➢ A newly occurring condition which influences the efficacy of the study investigation or is contra-indicative to the intake of study medication or which needs to be treated with a medication which is not permitted as a concurrent medication during the study
- ➢ Retroactive appraisal of either unfulfilled inclusion criteria or fulfilled exclusion criteria after the decision of the examining physician/leader of the clinical study
- ➢ Medically necessary transfer of the patient to a different department/a different hospital during the study phase
- ➢ Unexpected findings which make the continuation of therapy from an ethical or medical point of view unjustifiable. The decision will be made by the clinical examination leader

If the patient withdraws their consent the treating physician will end the patient’s participation in the study.

The complete study can be discontinued prematurely if it is perceptible early on that it cannot fulfill the aforementioned inclusion criteria.

This includes:

- ➢ The necessary recruiting numbers cannot be achieved
- ➢ There are serious violations of the protocol
- ➢ The documentation is incomplete or was deliberately filled out incorrectly and legal or ethical instructions are not met

Discontinuation due to reasons mentioned above is only possible if an agreement between the study leader, examining physicians and the biometric statistician can be reached.

In case of serious occurring AEs or severe side effects, or in case of accumulating AEs, the leader of the clinical investigation can terminate the study. In particular, the study will be stopped in case of undesirable results which question the safety of the homeopathic therapy.

### Adverse events and serious adverse events

Since this clinical examination is subject to formalities of a regulatory phase IV study and will be carried out in accordance to Good Clinical Practice (GCP) guidelines, any adverse event (AE) and serious adverse event (SAE) observed during the trial will be reported.

Each AE will be assessed by the examining physician according to its severity and its possible connection to the investigated therapy. SAEs will be documented using the MedDRA® standard of reporting SAEs [31].

SAEs will immediately be reported to the responsible ethics commission, the responsible federal authority, the participating examiners as well as the biometrician and thus a benefit to risk profile will be outlined. Documentation and assessment will be done using the OPEN-CLINICA reporting system [32].

### Adherence to interventions

To enhance the validity of the data, participants have to return the unused medical vials with remaining globules after the last intervention day and the final visit. Unused vials with globules will be counted and recorded in the drug accountability log.

### Outcomes

The primary endpoint of this study is pain reduction as measured by the subjective pain sensation on a straightforward 100-mm Visual Analogue Scale (VAS). The VAS will be handed out to patients for their individual evaluation of their subjective pain perception. For each entry, a VAS scale is designated on a separate sheet, so patients will be able to fill in the scale independently. The pages will then be collected as source data and kept in each patient file. Measurement of pain will occur four times a day and the average will be determined for each day.

The secondary endpoint is the amount of post-surgical intake of analgesic / anti-inflammatory agents. The number and dosages (in milligrammes) of the medical application will be collected for standard as well as the individually requested medicine. All occurring AEs will be descriptively recorded in the Case Report Form (CRF). Data regarding medication requested and taken by the patient will be extracted from the medical record folder.

In order to be able to describe the post-operative pain more precisely the standardised ‘Pain Experience Scale’ (German: Schmerzempfindungsskala (SES)) will be applied during study visits T− 1, T1, T3 and T5 once a day (see also Fig. 2 and Additional file 1: Standard Protocol Items: Recommendations for Interventional Trials (SPIRIT) Checklist). Due to their self-explanatory character, supervision in completing the VAS and SES will not be necessary.

### Randomisation

Participants will be randomised by a statistician on a 1:1 ratio using a computer-generated, random allocation sequence. The randomisation list will be kept strictly confidential.

### Allocation

The homeopathic medicine and placebo are provided by the Deutsche Homöopathie-Union (DHU) as bulk product (Reg. Nr. 2,502,726 for the study drug and Mat. Nr. 500,003,033 for the placebo). Both will be repacked in glass bottles and labelled according to the randomisation list at the pharmacy of the Community Hospital Herdecke and allocated to the particular patient by the associated study nurse. The identification will be carried out according to a uniform, identical sticker according to the GCP ordinance in obligatory labelling of testing compounds according to § 5 where for information only the continuing identification number will be noted (GCP-V § 5).

### Blinding

Participants, the principal investigator and the subinvestigator, the study nurse and the statistician will remain blinded to the identity of the two treatment groups until the end of the study.

Unblinding of individual participants through the investigator occurs in cases of SAEs if there is a sufficient causal link to the trial medication. In case of emergency, envelopes are deposited in the Investigator Site File (ISF) in order to clarify the random allocation.

### Sample size calculation

In accordance to the usual standard deviations of the 20-mm VAS, a minimal relevant difference of 15 mm and a power of 0.90, the level of significance for the testing of pain reduction at a significance level of α = 0.05 requires a minimum sample size of *n* = 40 in each group is aimed for the two-sided *t* test in the analysis of covariance (ANCOVA) model. Assuming a moderate correlation of *ρ* of between 0.2 and 0.5 [33] and under the assumption of a non-compliance of 20–25%, a total of *n* = 50 patients per group is sufficient.

### Data collection and management

**Fig. 1:**
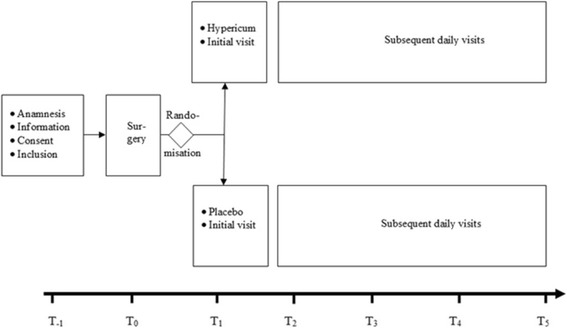
Flow chart of the course of the study

**Fig. 2:**
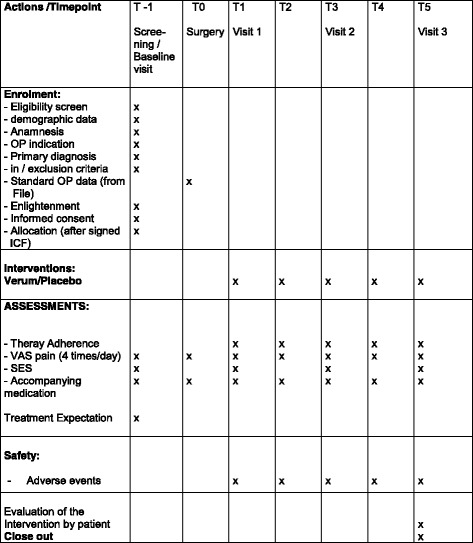
Schedule of enrolment, interventions and assessments

Study data will be collected from day − 1 until day 5 (Fig. 1). Data will be collected from medical records, scales and questionnaires and then transferred into the standardised paper CRFs designed for the study (Fig. 2). The original source data from the CRFs will be entered into a database using OPEN-CLINICA as the study documentation software [32].

CRFs will be stored in numerical order in a secure place and manner. Patient files will be maintained in storage for a period of 10 years after completion of the study. All forms related to the study data will be kept in locked cabinets. Access to the study data will be restricted.

### Statistical analysis

The statistical analysis of the study will be carried out at the Chair of Research Methodology and Statistics in Psychology, Witten/Herdecke University, using SAS as validated software for clinical studies [34].

### Populations for evaluation

All evaluations, in particular the evaluation of the primary outcome measure, will be made on the basis of all randomised patients (intention-to-treat population), regardless of whether or not they adhered to the treatment protocol or provided complete data sets. These are, in particular, patients:

- ➢ Who discontinued the clinical trial; they will be evaluated as if they had complied with it
- ➢ Who had their examining therapy modified or changed; they will be analyzed in their original randomised group
- ➢ Who took accompanying medication or received accompanying therapy without permission; they will still be taken into consideration in the analysis
- ➢ Whose planned examinations were not carried out within the planned time frame; they will still be taken into consideration in the analysis

Aside from the analysis of all randomised patients, a per-protocol analysis will also be carried out which is supposed to indicate which effect sizes can be reached under optimal circumstances. In the per-protocol population all patients will be included who fulfill all study requirements.

Patients who withdraw their consent to use their personal data for statistical analyses will be excluded from the analysis (dropouts).

### Dealing with missing values

Missing values will be replaced by applying the following measures:

- ➢ Missing reports of individual responses on the SES questionnaire will be replaced according to the recommendations of the Test Manual
- ➢ Missing total scores will be replaced numerous times according to the principle of multiple imputations (MI) by using the MCMC method [35]. The MI procedure assumes constant, multivariate, normally distributed data. A further requirement is the assumption of values missing at random. This does not additionally correlate with the actual size of the value which is to be replaced
- ➢ The replacement of missing values will be done with a model of regression model. It includes the final score as dependent variable and baseline values, study group membership and attitude of expectation as independent variables. A total of 50 complete data sets will be produced that way. From the 50 individual results point estimates will then be determined for the main objective and its variance

A detailed description about patient evolution within each group as well as the handling of non-compliant cases and missing values will be given in a future Statistical Analysis Plan according to [36] as a free update to this publication.

### Analysis of efficacy parameters

Merely the criteria of the primary objective will be evaluated inductively. All secondary outcome variables will be analyzed descriptively throughout the distribution across both therapy arms. For criteria that are of side objective, 95% confidence intervals and *p* values will be reported which are based on analogue models like the one for the main objective criterion. Those have a self-explanatory character and do not distinguish between the efficacies of the examined therapies.

### Analysis of main objective criterion

A univariate analysis of covariance (ANCOVA) will be used to generate results for the criterion of the main objective, which was to test whether there would be changes in pain intensity comparing to the beginning of the study to the fifth post-operative day on a 100-mm VAS. For this analysis the main parameter will be measured as a function of group affiliation (graded as a fixed factor on two levels), the baseline measure (linear, fixed covariate), patient expectation (ordinal, fixed factor on five levels), time and duration of surgery (as measured in hours) as well as pain intensity on the day before surgery will be modelled. Within this ANCOVA a *t* test, with a two-sided level of significance of α = 5%, will test whether both intervention groups differ from one another.

Additionally, the progress of VAS changes will be modelled with the help of a mixed linear model. For this model aside from group membership (binary), the VAS score at baseline (linear), time of surgery and patient expectation will be entered as fixed factors and the patient code (graded) will be entered as a random factor. The intra-individual correlation will be assumed to be exponentially decreasing with time. The estimated therapy effect, *p* values and confidence intervals will be based on the relevant *t* test.

### Auditing

The Center for Clinical Studies at Witten/Herdecke University (ZKS UW/H) will monitor and examine abidance with the study protocol.

### Confidentiality

The relevant regulations of the data protection legislation will be entirely fulfilled. All appropriate and necessary precautionary measures will be met in order to perpetuate the confidentiality of medical data and personal information.

### Regulatory and ethical approval

The application has been approved to the competent ethics committee (Witten/Herdecke University) and the Federal Institute for Drugs and Medical Devices (Bundesinstitut für Arzneimittel und Medizinprodukte, BfArM, Bonn, Germany).

This study is in compliance with the Helsinki Declaration and with the International Conference on Harmonisation (ICH) – Good Clinical Practice.

In case of necessary protocol amendments the amendment will be submitted to the ethics committee and competent authority and implementation will be done after approval. Due to the study design (monocentric, investigator-initiated trial) and close contact between study team, sponsor and site, a separate communication plan is not necessary.

## Discussion

Despite the fact that homeopathy has a long tradition in the complementary treatment of patients and is part of the medical curriculum in many European universities [37] there is an ongoing debate on its effectiveness and credibility. Thus, more robust clinical studies with clear and relevant endpoints are needed to substantiate the evidence base from primary to critical care.

This study evaluates the application of highly diluted *Hypericum* in a randomised controlled trial compared to placebo for the first time. The prescription of homeopathic *Hypericum* for pain conditions is historically well established and respective publications can be traced back to the medieval age [38]. In the field of homeopathy, Kent, the founder of American homeopathy, in his lectures on homeopathic Materia Medica states: ‘One who makes a study of the proving of *Hypericum* will be reminded of a class of injuries involving sentient nerves and it is not surprising that this remedy has come into use for the results of such injuries’ [39].

Neural pain after surgery caused by damage or injuries of the nerves often develops into stabbing and unbearable pain. Management of such post-surgical pain conventionally includes the application of opioids in varying dosage, codeine, dihydrocodeine, tilidine, naloxone or tramadol. If necessary, opioids are combined with peripheral pain-relieving medication or substances like gabapentin or pregabalin. As opioids are associated with a variety of potential side effects, including respiratory depression, homeopathic *Hypericum* is a promising additional treatment option in multimodal pain management, even though its effectiveness still has an insufficient evidence base [38]. Our study, therefore, may be seen as a first step towards providing clinical evidence. To ensure the quality of the study, its reliability and validity, the study protocol is based on the Consolidated Standards of Reporting Trials (CONSORT) guidelines and the SPIRIT guidance for clinical trials [40, 41]. If necessary, CONSORT extensions (i.e. for pragmatic randomised trials) will be considered in the final reporting of the study. Moreover, the checklists of Bornhöft et al. 2006 [42] and Mathie [43] for model validity of homeopathic treatment (MVHT) have been applied to obtain a broad acceptance of the study results.

This study makes use of a non-individualised classical homeopathic approach. Other researchers decided to make use of a complex homeopathic approach, i.e. in the treatment of low back pain due to osteoarthritis [44] or an individualised approach, i.e. in the pain management in osteoarthritis of the knee [45].

Our decision to apply only one remedy is based on the concept of ‘homeopathically proven indications’ where injuries are treated with typical remedies that were shown to be effective irrespective of the individuality of a given patient [17]. As *Hypericum* is known as a remedy for stabbing pain, especially when nerve root irritation or nerve damage is involved, we decided to apply *Hypericum* C200 as a sole remedy for this condition.

### Study limitations and bias

This is a monocentric study in an integrative hospital. Although the treatment completely adheres to the respective guidelines, the setting might not be regarded as representative for inpatient treatment of lumbar disc herniation.

### Trial status

At the time of initial manuscript submission, recruitment had already started (November 2015) but was not completed. The manuscript reports protocol version 2 (1 December 2016).

## Additional file

Additional file 1: SPIRIT 2013 Checklist: recommended items to address in a clinical trial protocol and related documents. (DOC 122 kb)
